# The *EPHA2* gene is associated with cataracts linked to chromosome 1p

**Published:** 2008-11-12

**Authors:** Alan Shiels, Thomas M. Bennett, Harry L.S. Knopf, Giovanni Maraini, Anren Li, Xiaodong Jiao, J. Fielding Hejtmancik

**Affiliations:** 1 Department of Ophthalmology and Visual Sciences, Washington University School of Medicine, St. Louis, MO; 2 Department of Genetics, Washington University School of Medicine, St. Louis, MO; 3 Department of Ophthalmology, University of Parma, Parma, Italy; 4 Ophthalmic Genetics and Visual Function Branch, National Eye Institute, National Institutes of Health, Bethesda, MD

## Abstract

**Purpose:**

Cataracts are a clinically and genetically heterogeneous disorder affecting the ocular lens, and the leading cause of treatable vision loss and blindness worldwide. Here we identify a novel gene linked with a rare autosomal dominant form of childhood cataracts segregating in a four generation pedigree, and further show that this gene is likely associated with much more common forms of age-related cataracts in a case-control cohort.

**Methods:**

Genomic DNA was prepared from blood leukocytes, and genotyping was performed by means of single nucleotide polymorphism (SNP) markers, and short tandem repeat (STR) markers. Linkage analyses were performed with the GeneHunter and MLINK programs, and association analyses were performed with the Haploview and Exemplar programs. Mutation detection was achieved by PCR amplification of exons and di-deoxy cycle-sequencing.

**Results:**

Genome-wide linkage analysis with SNP markers, identified a likely disease-haplotype interval on chromosome 1p (rs707455-[~10 Mb]-rs477558). Linkage to chromosome 1p was confirmed using STR markers D1S2672 (LOD score [Z]=3.56, recombination distance [θ]=0), and D1S2697 (Z=2.92, θ=0). Mutation profiling of positional-candidate genes detected a heterozygous transversion (c.2842G>T) in exon 17 of the gene coding for Eph-receptor type-A2 (*EPHA2*) that cosegregated with the disease. This missense change was predicted to result in the non-conservative substitution of a tryptophan residue for a phylogenetically conserved glycine residue at codon 948 (p.G948W), within a conserved cytoplasmic domain of the receptor. Candidate gene association analysis further identified SNPs in the *EPHA2* region of chromosome 1p that were suggestively associated with age-related cataracts (p=0.007 for cortical cataracts, and p=0.01 for cortical and/or nuclear cataracts).

**Conclusions:**

These data provide the first evidence that *EPHA2*, which functions in the Eph-ephrin bidirectional signaling pathway of mammalian cells, plays a vital role in maintaining lens transparency.

## Introduction

Light scattering opacities of the crystalline lens, or cataracts, are a common ocular symptom of aging (>50 years), and despite advances in their surgical treatment age-related cataracts remain a leading cause of visual impairment, accounting for ~40% of world blindness [1]. Rarely, cataracts may present at birth (congenital), during infancy or during childhood with an estimated prevalence of 1–13 cases/10,000 live births [2], and are clinically important as a cause of impaired form vision development accounting for 5%–20% of childhood blindness [3]. As much as 40% of early-onset cataracts may have a genetic basis [2], either as a recognized feature of over 50 genetic syndromes, involving other ocular defects (e.g., microphthalmia) and systemic abnormalities (e.g., galactokinase deficiency), or more often as an isolated lens phenotype with autosomal dominant inheritance (Online Mendelian Inheritance in Man; OMIM). To date, genetic linkage studies of over 70 extended families worldwide have mapped at least 25 independent loci for clinically diverse forms of nonsyndromic cataracts on 18 human chromosomes [4]. The majority of underlying mutations have been identified in 10 crystallin genes (*CRYAA*, *CRYAB*, *CRYBB1*, *CRYBB2*, *CRYBB3*, *CRYBA3/A1*, *CRYBA4*, *CRYGC*, *GRYGD*, and *CRYGS*) [5-13] and 2 connexin genes (*GJA3*, *GJA8*) [14,15], which encode the major “refractive” proteins and gap-junction-channel proteins of the lens, respectively. The remaining mutations have been identified in at least 10 other functionally diverse genes including those coding for transcription factors (*HSF4*, *MAF*, *PITX3*) [16-18], an aquaporin water channel (*MIP*) [19], cell-junction-like proteins (*LIM2*, *TMEM114*) [20,21], intermediate-filament-like proteins (*BFSP2*, *BFSP1*) [22,23], a chromatin modifying protein (*CHMP4B*) [24], and a solute carrier protein (*SLC16A12*) [25].

In addition to conventional linkage studies of inherited cataracts in extended families, sibling and twin studies indicate that genetic risk factors may account for 14%–48% of the heritability for age-related “nuclear” cataracts [26-28], and 24%–75% of the heritability for age-related “cortical” cataracts [29-31]. In contrast to inherited cataracts; however, comparatively little is known about the underlying genetic determinants predisposing to age-related cataracts [32]. Genes linked with inherited forms of cataracts represent likely candidate genes for age-related cataracts; however, only the “Osaka” variation (p.A198V) in the gene for autosomal recessive galactokinase-deficiency and congenital cataracts (*GALK1*) has so far been associated unambiguously with age-related cataracts, and this association appears to be limited to East Asian populations [33,34]. Here we report for the first time that variations in the gene for Eph-receptor tyrosine kinase-type A2 (*EPHA2*) on 1p36 are associated with inherited and age-related forms of cataracts in Caucasians.

## Methods

### Family participants

A white American family of Western European origin (family Mu) was ascertained through ophthalmic records in the Department of Ophthalmology and Visual Sciences at Washington University School of Medicine, St. Louis, MO. Blood samples were obtained from 18 family members including 12 affected individuals, one unaffected individual, and five spouses. Leukocyte genomic DNA was purified using the Gentra Puregene Blood kit (Qiagen, Valencia, CA), and quantified by absorbance at 260nm (GeneQuant *pro*; GE Healthcare, Waukesha, WI). Ethical approval for this study was obtained from the Washington University Human Research Protection Office, and written informed consent was provided by all participants before enrollment in accordance with the tenets of the Declaration of Helsinki, and Health Insurance Portability and Accountability Act (HIPAA) regulations.

### Case-control participants

A case-control cohort of unrelated individuals aged >50 years form Northern Italy was ascertained from the Clinical Trial of Nutritional Supplements and Age-Related Cataract Study [35]. Genomic DNA was purified from blood samples as described previously [34], and lens status was evaluated by grading slit-lamp and retro-illumination lens photographs according to a modification of the Age-Related Eye Disease Study (AREDS) cataract grading system [36]. Briefly, a clear lens was defined by the following grades at the last available grading: nuclear opalescence <3.0; cortical opacity <5% within the five mm diameter (dia) circle at the center of the pupil, and <15% outside this central five mm dia circle; posterior sub-capsular opacity <1.0 % within the five mm dia central circle. A lens was defined as cataractous when at least one type of opacity was present with one or more of the following grades: nuclear opalescence ≥4.5; cortical opacity >25% inside five mm dia central circle and/or >25% outside five mm dia circle; posterior sub-capsular opacity >12.5% inside five mm dia circle. Ethical approval for this study was obtained from the University of Parma and the National Eye Institute, and written informed consent was provided in accordance with the tenets of the Declaration of Helsinki.

### SNP genotyping and linkage analysis

For genome-wide linkage analysis, genotyping was performed by means of the HumanLinkage-12 Genotyping Beadchip and the Infinium-II whole-genome amplification and single-base extension assay (Illumina, San Diego CA) in the Microarray Core Facility at Washington University Genome Center. Parametric multipoint linkage analysis performed with GeneHunter version 2.1r5 from the easyLINKAGE Plus version 5.08 package [37]. SNP marker allele frequencies used for linkage analysis were those calculated for Caucasians by the HapMap project. A gene frequency of 0.001 and a penetrance of 100% were assumed for the disease locus. Computation was performed with GeneHunter in sets of 100 markers.

### STR genotyping and linkage analysis

STR markers from the National Center for Biotechnology Information (NCBI) combined Généthon, Marshfield, and deCODE genetic linkage maps were genotyped by means of a 4200 DNA analyzer running Gene ImagIR software (Li-Cor, Lincoln, NE) as described previously [38]. Pedigree and haplotype data were managed using Cyrillic (v.2.1) software (FamilyGenetix Ltd., Reading, UK), and two-point LOD scores (Z) calculated using the MLINK sub-program from the LINKAGE (5.1) package of programs [39]. Marker allele frequencies were assumed to be equal, and a gene frequency of 0.0001 with a penetrance of 100% were assumed for the disease locus.

### Mutation analysis

The genomic sequence for *EPHA2* was obtained from the Ensembl human genome browser, and gene-specific M13-tailed PCR primers (Table 1) were selected from the NCBI re-sequencing amplicon (RSA) probe database. Genomic DNA (2.5 ng/ul, 20 ul reactions), was amplified (35–40 cycles) in a GeneAmp 9700 thermal cycler using AmpliTaq polymerase (Applied Biosystems, Foster City, CA) and gene-specific primers (10 pmol). Resulting PCR amplicons (~250–650 bp) were either enzyme-purified with ExoSAP-IT (USB Corporation, Cleveland, OH) or gel-purified with the QIAquick gel-extraction kit (Qiagen). Purified amplicons were direct cycle-sequenced in both directions with BigDye Terminator Ready Reaction Mix (version 3.1) containing M13 forward or reverse sequencing primers then ethanol precipitated and detected by capillary electrophoresis on a 3130xl Genetic Analyzer running Sequence Analysis (version 5.2) software (Applied Biosystems), and Chromas (version 2.23) software (Technelysium, Tewantin, Queensland, Australia). For allele-specific PCR analysis, exon-17 was amplified with three primers (Ex17R1, Ex17SF, and T-alleleF; Table 1), and resulting amplicons were visualized at 302 nm following electrophoresis in 3% agarose-gels stained with GelRed (Biotium, Hayward, CA).

**Table 1:** PCR primers for mutation-profiling of *EPHA2*.

**Primer**	**Location**	**Strand**	**Sequence (5′-3′)**
Ex1F	Intron-1	Antisense	gcgaccaagctgaaaccgcttatt
Ex1R	5′-upstream	Sense	ggcatgaatgaacaggagtcggtt
Ex2F	Intron-2	Antisense	cgtaccttcccacgcccatc
Ex2R	Intron-1	Sense	ccagcctgctgtgtgccttc
Ex3F1	Exon-3	Antisense	ACGGCATGGTCCACACAGGT
Ex3R1	Intron-2	Sense	ccaggcacctgcccacacta
Ex3F2	Intron-3	Antisense	aaggaaactgatgtctgggaagga
Ex3R2	Exon-3	Sense	CCAGAAGCGCCTGTTCACCA
Ex4F	Exon-5	Antisense	CAGACTCGGGCCAGCACTGT
Ex4R	Intron-3	Sense	ttcctgggtgcccggtacat
Ex5F	Intron-5	Antisense	gacactgtgcctttaaccacttgctc
Ex5R	Exon-4	Sense	GCTTCTTCCGGGCACCTCAG
Ex6F	Intron-6	Antisense	ctctgctgtgctgccttggg
Ex6R	Intron-5	Sense	tgcctgctcgtaggcagctt
Ex7F	Intron-7	Antisense	ccgccggtgaccgagaaag
Ex7R	Intron-6	Sense	ggcgtccttggaagaggcag
Ex8F	Intron-8	Antisense	gattcctgcctgcgtgtcca
Ex8R	Intron-7	Sense	tggagccttcccaagtgcaa
Ex9/10F	Intron-10	Antisense	accaccgctgcctcctca
Ex9/10R	Intron-8	Sense	tgggccgcattctgagcac
Ex10/11F	Intron-11	Antisense	cctctccacccagtgtgggc
Ex10/11R	Intron-9	Sense	cccacagcctggtccaagtc
Ex12F	Exon-13	Antisense	TGGTGGTGTAGGTGGCCTCG
Ex12R	Intron-11	Sense	tacctctgcccactcctccg
Ex13F	Exon-14	Antisense	CGTCGCTGGCAGAGGTGAAC
Ex13R	Exon-12	Sense	CCCTGGACAAGTTCCTTCGGg
Ex14F	Intron-14	Antisense	aactgtcctctgcccagccc
Ex14R	Exon-13	Sense	CGAGGCCACCTACACCACCA
Ex15F	Intron-15	Antisense	ctgggccatcgtgtccagtc
Ex15R	Intron-14	Sense	gggcagctctgaaggttggg
Ex16F	Intron-16	Antisense	tggcggagttctgcccttct
Ex16R	Intron-15	Sense	gactgggcttccctgttgcc
Ex17F1	Exon-17	Antisense	agggaccgctttgggtctca
Ex17R1	Intron-16	Sense	ctctccctctctccctcccg
Ex17F2	Exon-17	Antisense	ccctgccacacacacacattc
Ex17R2	Exon-17	Sense	agtggcctccctgctgtgc
Ex17F3	3′-downstream	Antisense	gctcccagggttaagtgacgtg
Ex17R3	Exon-17	Sense	gcagactgtgaacttgactgggtga
T-alleleF	Exon-17	Antisense	GCCGGGCAGCCGCACCCA
Ex17SF	Exon-17	Antisense	ggaggccactctgtttcttcaagt

Gene specific primers used for amplification and sequencing of exons 1–17 and flanking intron regions of *EPHA2* on chromosome 1p. Coding and noncoding sequence are shown in uppercase and lowercase, respectively. Antisense primers were 5′-tailed with the M13-forward universal sequence (5′-tgtaaaacgacggccagt), and sense primers were 5′-tailed with the M13-reverse universal sequence (5′-caggaaacagctatgacc). The allele-specific primer (T-alleleF) was used to detect the heterozygous c.2842G>T transversion in exon-17 as shown in Figure 3.

### SNP association analysis

For association studies, tagging SNPs covering the *EPHA2* region were selected from the HapMap database using the Haploview program [40]. SNPs were genotyped by a combination of TaqMan melt-curve assays “on-demand” (Applied Biosystems), SNaPshot single-base extension assays (Applied Biosystems), and direct BigDye Terminator cycle-sequencing (Applied Biosystems). TaqMan assays were run on a 7900 HT real-time PCR system (Applied Biosystems). SNaPshot assays and di-deoxy cycle-sequencing were performed on a 3130 DNA analyzer (Applied Biosystems), and sequence analysis was performed with the Mutation Surveyor program (Soft Genetics, State College, PA).

### Statistical analyses

Tagging SNPs and haplotype blocks were identified using the Haploview program [40], and association statistics including χ^2^, trend p values, and odds ratios were calculated using the Exemplar program version 4.04 (Sapio Sciences, York, PA). Hardy–Weinberg equilibrium was assessed using a χ^2^ test implemented in the Exemplar program.

## Results

### Linkage studies

**Figure 1:**
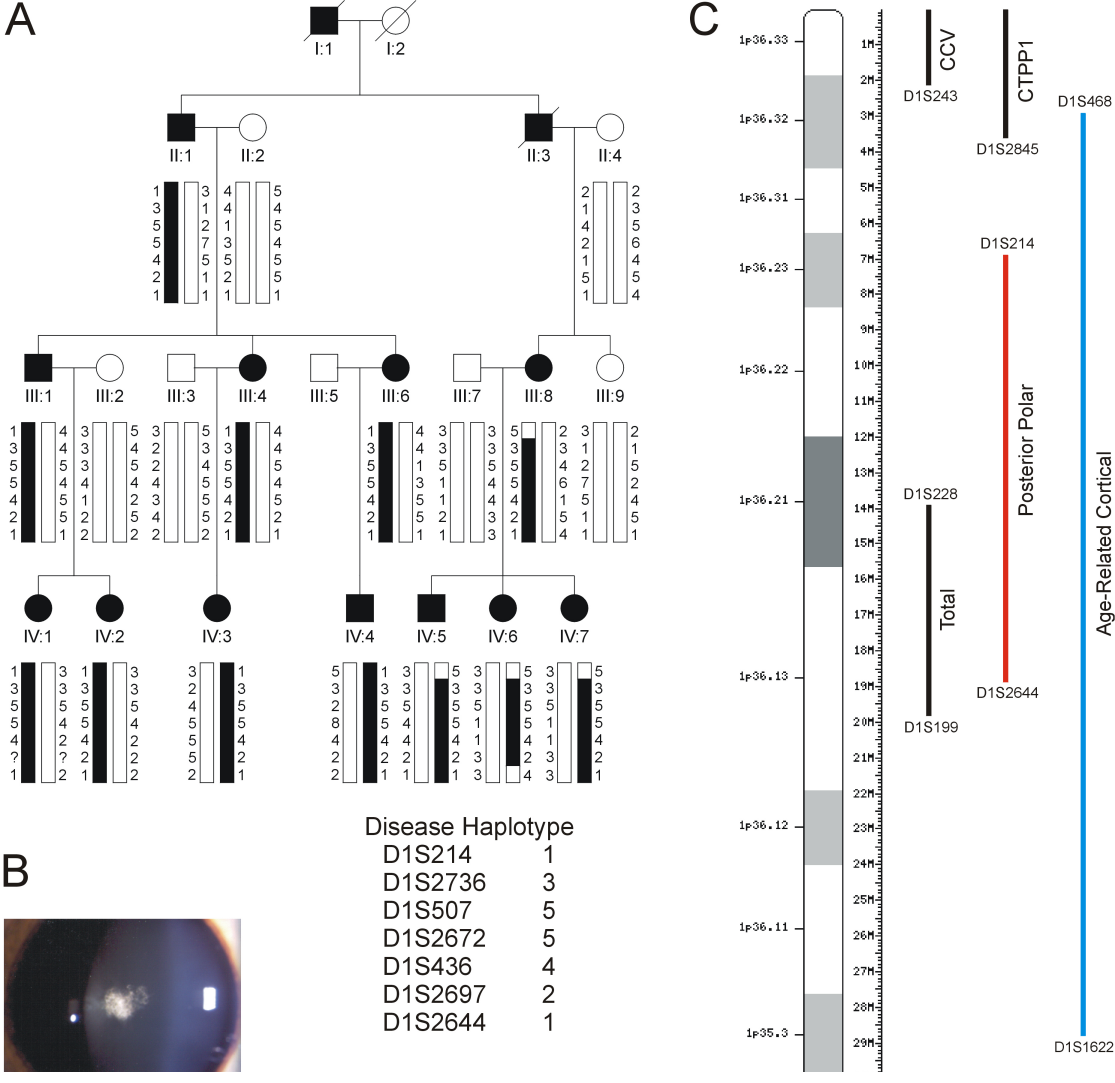
Autosomal dominant posterior polar cataracts in a four generation white American pedigree (family Mu). **A:** Pedigree and haplotype analysis showing segregation of seven STR markers on chromosome 1p listed in descending order from the telomere. Squares and circles denote males and females, respectively. Filled symbols and bars denote affected status and haplotypes, respectively. **B**: Slit-lamp image of left lens from affected female IV:6 (age 12 years) showing posterior sub-capsular  opacity. **C**: Ideogram of chromosome 1p36, comparing the cytogenetic and physical locations of STR markers defining the posterior polar cataract locus in this study (red) with those defining 3 other loci (black) for autosomal dominant cataracts (CCV, CTPP1 and Total) [45-47], and a locus (blue) for age-related cortical cataracts [44]. M, mega-base pairs.

We investigated a four generation white family from the United States (family Mu) segregating “posterior polar” cataracts in the absence of systemic abnormalities (Figure 1A). Autosomal dominant inheritance was supported by father-to-son transmission in the absence of gender bias or skipping of generations. Ophthalmic records indicated that the cataracts usually presented in both eyes as disc-shaped posterior sub-capsular opacities with evidence of posterior lenticonus (Figure 1B). In three affected individuals, opacification progressed to affect the central (nucleus) and anterior polar regions of the lens (IV:5, IV:6, IV:7; Figure 1A). In addition to cataracts, one affected individual had monocular amblyopia (III:8), and two others developed strabismus requiring corrective surgery (IV:1, IV:5). The age-at-diagnosis varied from birth to15 years, and the age-at-surgery ranged from 0 to 44 years. Post-surgical corrected visual acuity varied from 20/20 to 20/70 in the better eye.

**Figure 2:**
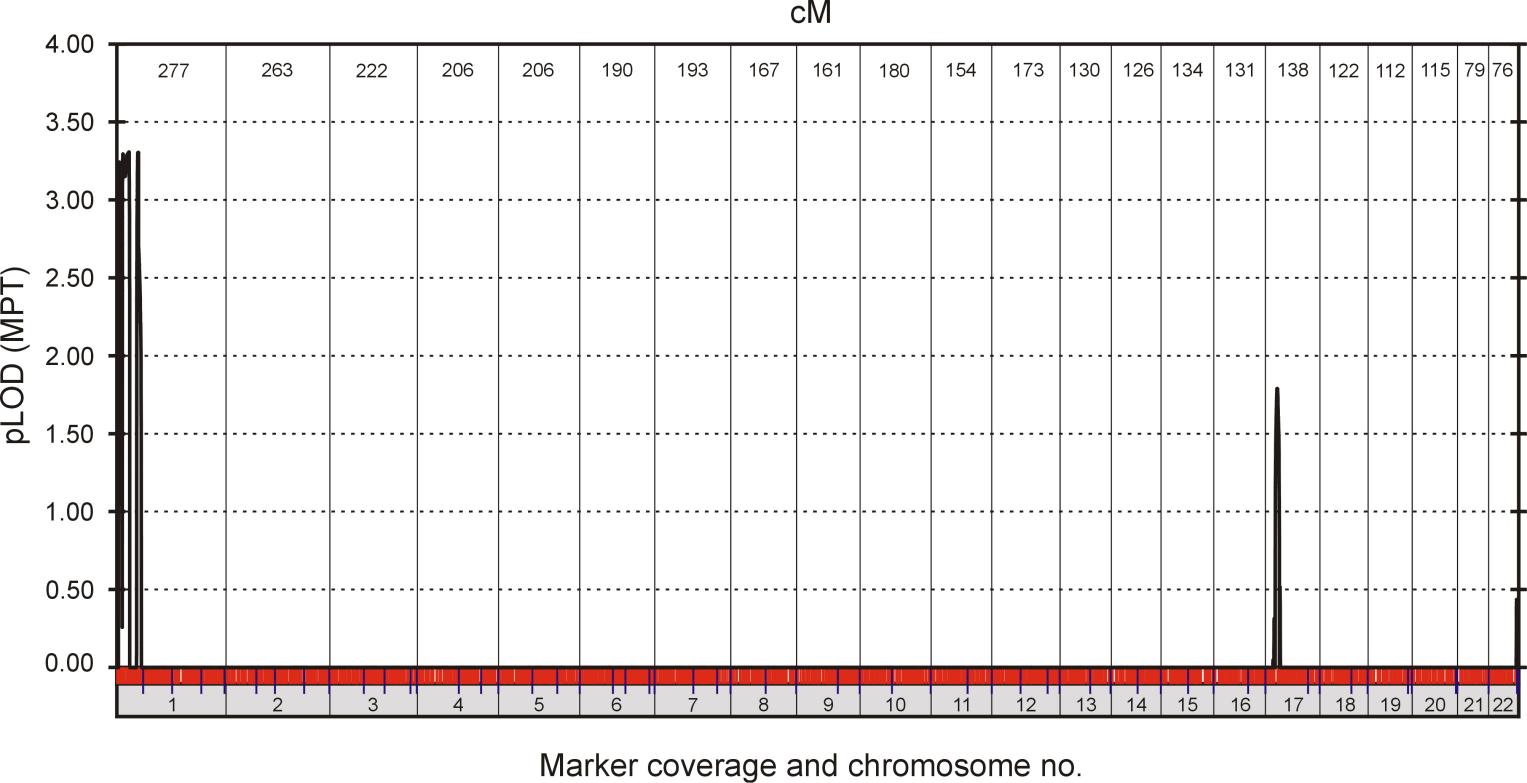
Parametric multipoint LOD scores (pLOD) for linkage between the posterior polar cataract phenotype in family Mu and SNP markers across the 22 autosomes.

For genome-wide linkage analysis, the 12 affected individuals in family Mu were genotyped by means of the HumanLinkage-12 Panel (Illumina), which comprises 6090 SNP markers uniformly spaced at an average genetic distance of 0.58 cM. Parametric multipoint analysis performed with GeneHunter (v2.1r5) detected significant evidence of linkage (Figure 2) on chromosome 1p (pLOD 3.3), and weak evidence of linkage on chromosome 17p (pLOD 1.8). Haplotype reconstruction further identified a common disease interval on 1p36 (rs707455-[~10Mb]-rs477558) that cosegregated with the disease in all 12 affected individuals (Table 2).

**Table 2:** SNP disease haplotype for the cataract-linked interval on chromosome 1p.

**SNP**	**Disease allele**	**Distance from 1-ptel**	
		**Mb**	**cM**
rs707455 *	C	7.75	13.68
rs8627	A	8.34	14.21
rs1473420	A	8.54	14.48
rs916380	A	9.26	15.43
rs630075	T	9.29	15.47
rs6677649	A	9.67	16.64
rs2119508	G	9.78	16.66
rs2379107	A	9.83	16.67
rs7528979	C	10.02	16.70
rs6541085	A	10.21	16.73
rs912962	T	10.27	16.74
rs649101	T	10.41	16.77
rs2506887	A	10.51	16.78
rs481453	T	10.60	16.99
rs488834	T	10.69	17.33
rs7555879	G	10.81	17.76
rs2273348	C	11.00	18.43
rs4846012	G	11.48	20.07
rs2817594	A	11.54	20.24
rs3818157	G	11.95	21.26
rs235256	C	12.22	21.31
rs761162	A	14.24	24.73
rs1416633	C	14.36	24.96
rs7531416	A	14.62	25.58
rs3927648	A	14.74	25.86
rs1883661	A	14.88	26.20
rs4357518	A	14.95	26.46
rs3845596	T	15.06	26.91
rs7515649	C	15.31	27.49
rs488595	G	15.56	27.95
rs1007150	T	16.49	29.61
rs761423	G	17.17	30.83
rs1005753	A	17.32	31.08
rs3766306	T	17.84	32.02
rs1578215	G	17.99	32.31
rs477558 **	G	18.09	32.53

The disease associated allele is shown for each SNP in the cataract interval, which spans ~10 megabases (Mb) or ~19 centi-Morgans (cM). The asterisk denotes critical recombinant individual III:8 in family Mu (Figure 1A) with genotype T/T at rs707455. The double asterisk denotes critical recombinant individual IV:6 (Figure 1A) with genotype A/A at rs477558.

To validate the authentic SNP interval from the genome-wide linkage scan, all 18 family members were genotyped with STR markers. We first excluded false-positive linkage to the SNP interval on 17p using markers D17S1852 (Z=-3.75, θ=0) and D17S921 (Z=-∞, θ=0, data not shown). Subsequently, we obtained confirmatory evidence of linkage on 1p (Table 3) for markers D1S2672 (Z=3.56, θ=0), D1S2697 (Z=2.92, θ=0), and D1S436 (Z=2.84, θ=0). Haplotype analysis detected four recombinant individuals within the Mu pedigree (Figure 1A). An affected female (III:8), her affected son (IV:5), and two affected daughters (IV:6, IV:7) were obligate recombinants distal to the centromere at D1S214. In addition, one of the affected daughters (IV:6) was recombinant proximal to the centromere at D1S2644. However, no further recombinant individuals were detected at five other intervening STR markers, consistent with the cataract locus residing in the physical interval, D1S214-(~12 Mb)-D1S2644 (Figure 1C), which was coincident with the SNP interval on 1p.

**Table 3:** Two-Point LOD scores (Z) for linkage between the cataract locus in family Mu and markers on chromosome 1p.

**Marker**	**Distance from 1p-tel**		**Z at θ=**						**Z_Max_**	**θ_Max_**
	**cM**	**Mb**	**0.00**	**0.05**	**0.10**	**0.20**	**0.30**	**0.40**		
D1S2795	14.20	5.50	-0.47	2.24	2.24	1.88	1.33	0.66	2.27	0.07
D1S214	16.40	6.88	-0.44	2.27	2.27	1.91	1.35	0.67	2.30	0.08
rs707455		7.75	-3.70	-0.64	-0.37	-0.15	-0.05	-0.01	0.00	0.05
D1S2736	22.90	10.54	2.62	2.38	2.14	1.62	1.05	0.47	2.62	0.00
D1S489	32.30	11.97	1.54	1.41	1.28	1.01	0.71	0.38	1.54	0.00
D1S507	36.20	14.90	2.61	2.38	2.13	1.60	1.03	0.45	2.61	0.00
D1S2672	36.20	15.15	3.56	3.25	2.93	2.25	1.51	0.71	3.56	0.00
D1S436	39.90	15.74	2.84	2.59	2.32	1.76	1.15	0.52	2.84	0.00
D1S2697	39.90	16.29	2.92	2.66	2.39	1.82	1.20	0.55	2.92	0.00
EPHA2 c.2842G>T		16.32–16.36	3.61	3.30	2.98	2.28	1.52	0.72	3.61	0.00
D1S1592		17.94	1.26	1.16	1.05	0.83	0.59	0.32	1.26	0.00
rs477558		18.09	-4.43	-0.57	-0.33	-0.14	-0.06	-0.02	0.00	0.50
D1S2644	46.20	18.90	-∞	-0.38	-0.15	0.03	0.07	0.06	0.07	0.32

Z values for markers on 1p listed in genetic and physical distances measured in cM and Mb, respectively, from the telomere (1p-tel). The maximum Z values (Zmax) occurred at marker D1S2672, and at the c.2842G>T transversion in exon-17 of *EPHA2*. θ=recombination fraction.

### Mutation analysis

**Figure 3:**
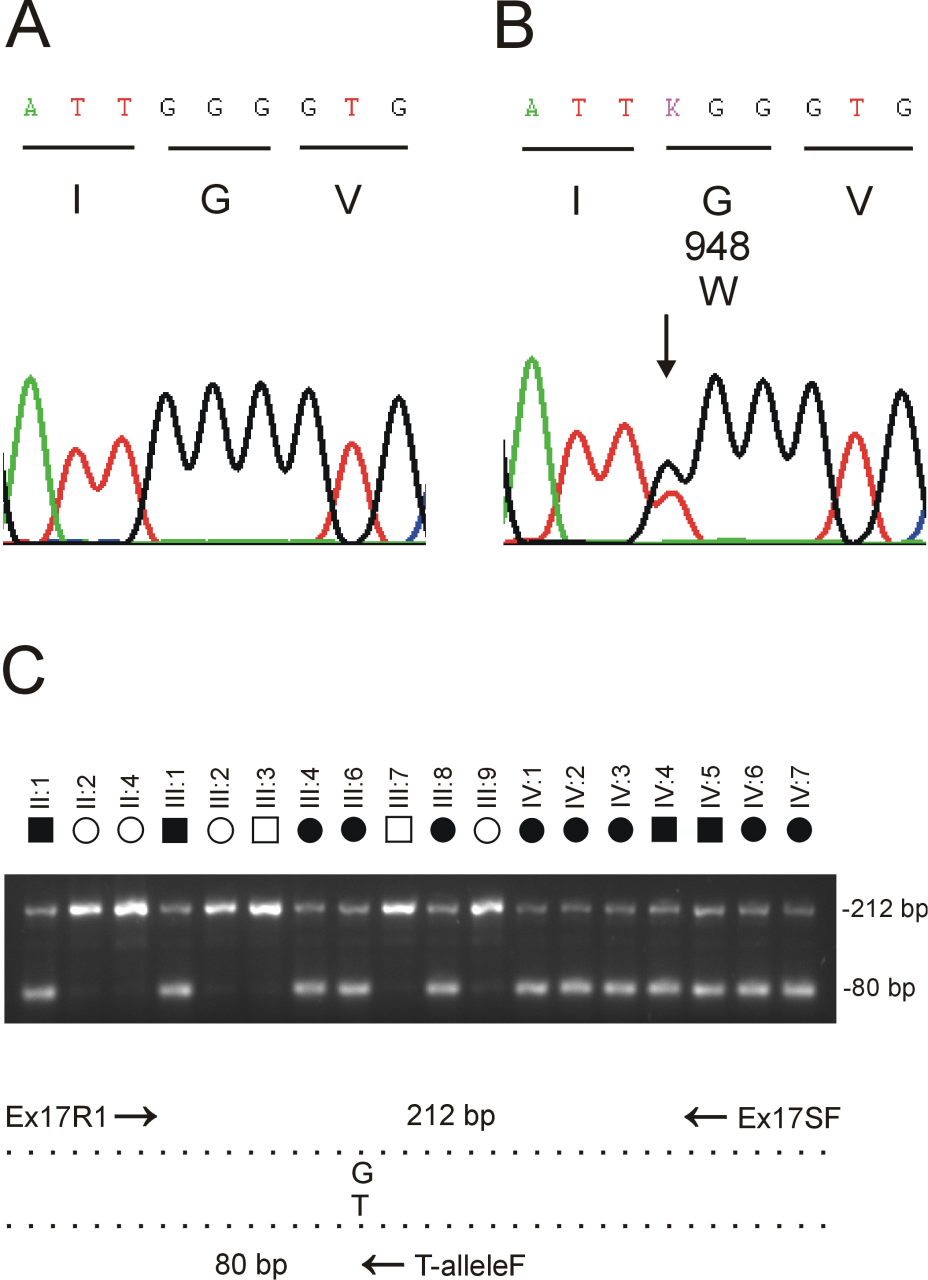
Mutation analysis of *EPHA2* in family Mu. **A**: Sequence trace of the wild type allele showing translation of glycine (G) at codon 948 (GGG). **B**: Sequence trace of the mutant allele showing the heterozygous c.2842G>T transversion (denoted K by the International Union of Pure and Applied Chemistry [IUPAC] code) that is predicted to result in the missense substitution of tryptophan (TGG) for glycine at codon 948 (p.G948W). **C**: Allele-specific PCR analysis using the 3 primers (Table 1) indicated by arrows in the schematic diagram; exon-17 was amplified as above with the sense (anchor) primer located in intron 16 (Ex17R1), the anti-sense primer located in the 3′-untranslated region (Ex17SF), and the nested mutant primer specific for the T-allele in codon 948 (T-alleleF). Note that only affected members of family Mu are heterozygous for the mutant T-allele (80 bp).

The SNP and STR intervals contained 211 and 219 positional-candidate genes, respectively, none of which were obvious functional candidates for cataracts in family Mu (NCBI Map Viewer). We prioritized genes for mutation analysis of exons and intron boundaries (splice-sites) primarily based on evidence of expression or function in the eye, from the NCBI UniGene expressed sequence tag (EST) database, and the NEIBank bioinformatics resource for vision research [41]. Re-sequencing analysis of individuals II:1, III:8, and III:9 from the Mu pedigree (Figure 1A) excluded the presence of coding or splice-site mutations in several genes including *ENO1* (GeneID: 2023), which according to NEIBank is abundantly expressed in the human lens. However, re-sequencing of a 17-exon gene symbolized *EPHA2* (GeneID: 1969), which is also expressed in human lens (NEIBank), identified a heterozygous c.2842G>T transversion in exon-17 that was not present in wild type (Figure 3B). This single nucleotide change did not result in the gain or loss of a convenient restriction site, therefore we designed allele-specific (G/T) PCR analysis to confirm that the mutant “T” allele cosegregated with affected but not unaffected members of family Mu (Figure 3C). Furthermore, when we tested the c.2842G>T transversion as a bi-allelic marker, with a notional allelic frequency of 1%, in a two-point LOD score analysis of the cataract locus (Table 3) we obtained further compelling evidence of linkage (Z=3.61, θ=0). In addition, we confirmed that the c.2842G>T transversion was not listed in the NCBI SNP database (dbSNP), and excluded it as a SNP in a panel of 192 normal unrelated individuals (i.e., 384 chromosomes) using the allele-specific PCR analysis described in Figure 3C (data not shown). While it is possible that an undetected mutation lay elsewhere within the disease-haplotype interval, our genotype data strongly suggested that the c.2842G>T transversion in exon 17 of *EPHA2* represented a causative mutation rather than a benign SNP in linkage disequilibrium with the cataract phenotype.

**Figure 4:**
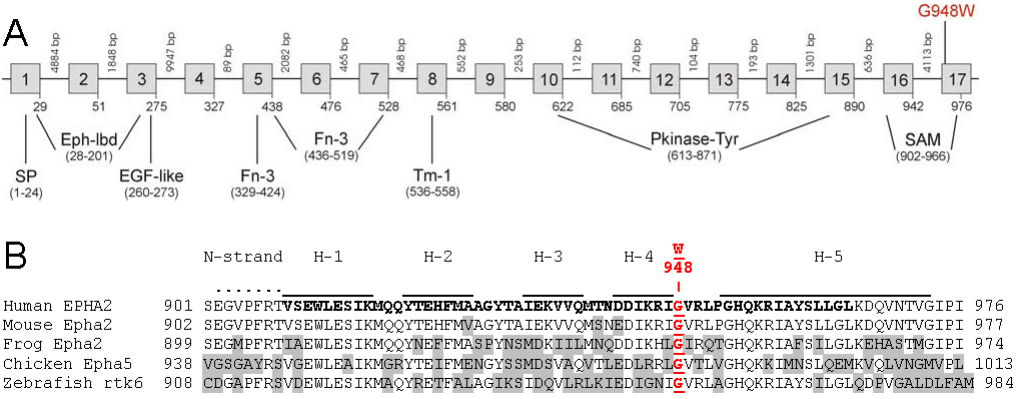
Schematic diagram showing gene structure and protein domains of *EPHA2*. **A**: Exon-intron organization, mutation profile and protein domains. Intron sizes are shown in base-pairs (bp), and codons are numbered below each exon (boxes). The approximate locations of protein domains are indicated. SP, signal-peptide; Eph-lbd, Eph-receptor ligand binding domain; EGF-like, epithelial growth factor-like region; Fn-3, fibronectin type-III domain; Tm-1, transmembrane domain type-1; Pkinase-Tyr, protein tyrosine kinase domain; SAM, sterile-α-motif. The predicted p.G948W missense mutation identified in family Mu is proposed to reside in the cytoplasmic SAM domain. **B**: Amino acid sequence alignment of the SAM domain from human EPHA2 and orthologs from other species, showing conservation of p.G948. Divergent amino-acids residues are shaded. The SAM domain comprises an NH_2_-terminal extended strand (dots), 5 α-helices (H1–5, over-lined), and a core sub-unit (bolded). The proposed p.G948W substitution likely resides between H-4 and H-5.

The c.2842G>T transversion identified above occurred at the first base of codon 948 (GGG >TGG), and was predicted to result in the missense substitution of glycine to tryptophan (p.G948W) at the level of translation (Figure 4), placing it in the cytoplasmic sterile-α-motif (SAM) domain of EPHA2 [42,43]. Cross-species alignment of the amino acid sequences for EPHA2 present in the Entrez protein database, performed by means of ClustalW multiple sequence alignment, revealed that p.G948 is evolutionarily conserved within the bony vertebrates (Figure 4B). Moreover, the predicted p.G948W substitution represented a nonconservative amino acid change, with the small neutral, polar side-group (-H) of glycine replaced by the much larger neutral, hydrophobic side-group (-CH_2_-C_8_ H_4_NH) of tryptophan. Position specific score matrix analysis (PSSM Viewer) revealed a marked decline in value from +6 to −6 (Table 4) confirming that the predicted p.G948W substitution occurred less frequently than expected in proteins with the conserved SAM domain (Conserved Domain Database [CDD] pfam00536) further raising the likelihood of functional consequences.

**Table 4:** Position specific score matrix (PSSM) analysis of the predicted p.G948W substitution in EPHA2.

**Codon**	**Amino acid**	**Side-chain**	**Charge**	**Potential H-bonds**	**Residue weight**	**Isoelectric point**	**Hydro-phobicity**	**PSSM value**
GGG	G (Gly)	-H	0	0	57	6.0	0.770	6
TGG	W (Trp)	-CH2-C8 H4NH	0	1	186	5.9	0.854	−6


Differences in the physico-chemical properties of glycine and tryptophan are consistent with a non-conservative substitution. The negative PSSM value indicates that tryptophan substitutes for glycine at position 948 less frequently than expected among proteins containing the conserved SAM domain.

### Association studies

*EPHA2* resides within a large interval (D1S468-[~25.5 Mb]-D1S1622) on chromosome 1p (Figure 1C) that was previously linked with age-related cortical cataracts in families and sib-pairs from the Beaver Dam Eye Study [44]. To investigate the possibility that *EPHA2* was associated with age-related cataracts, we performed candidate-gene SNP allele association studies in a case-control cohort from Northern Italy [34,35]. This clinically well defined cohort was of similar European ancestry to that of family Mu, and comprised 126 unrelated individuals with nuclear cataracts, 119 with cortical cataracts, and 104 unrelated controls with clear lenses (Table 5). Of the cases, 28 had both nuclear and cortical cataracts, three had nuclear and posterior sub-capsular cataracts, 10 had cortical and posterior sub-capsular cataracts, and four had nuclear, cortical, and posterior sub-capsular cataracts.

**Table 5:** Age, gender and lens status of the Italian case-control cohort of age-related cataracts.

**Lens status**	**Age range**	**Mean age (±2 SD)**	**Males (%)**	**Females (%)**	**Total**
Any cortical cataracts	57–86	75.6 (63.7–87.5)	57 (48)	62 (52)	119
Any nuclear cataracts	50–86	75.8 (63.0–88.6)	75 (60)	51 (40)	126
Any cataracts	50–86	75.7 (63.2–88.2)	114 (54)	99 (46)	213
Clear lenses	61–86	74.5 (64.6–84.5)	61 (59)	43 (41)	104

The cohort of unrelated individuals comprised a total of 213 age-related cataract cases and 104 clear lens controls, matched for age and gender. Cases with both nuclear and cortical cataracts (32) were counted in both the any cortical and any nuclear groups. Cases with any posterior sub-capsular cataracts (17) were not analyzed separately due to statistically insufficient numbers.

**Figure 5:**
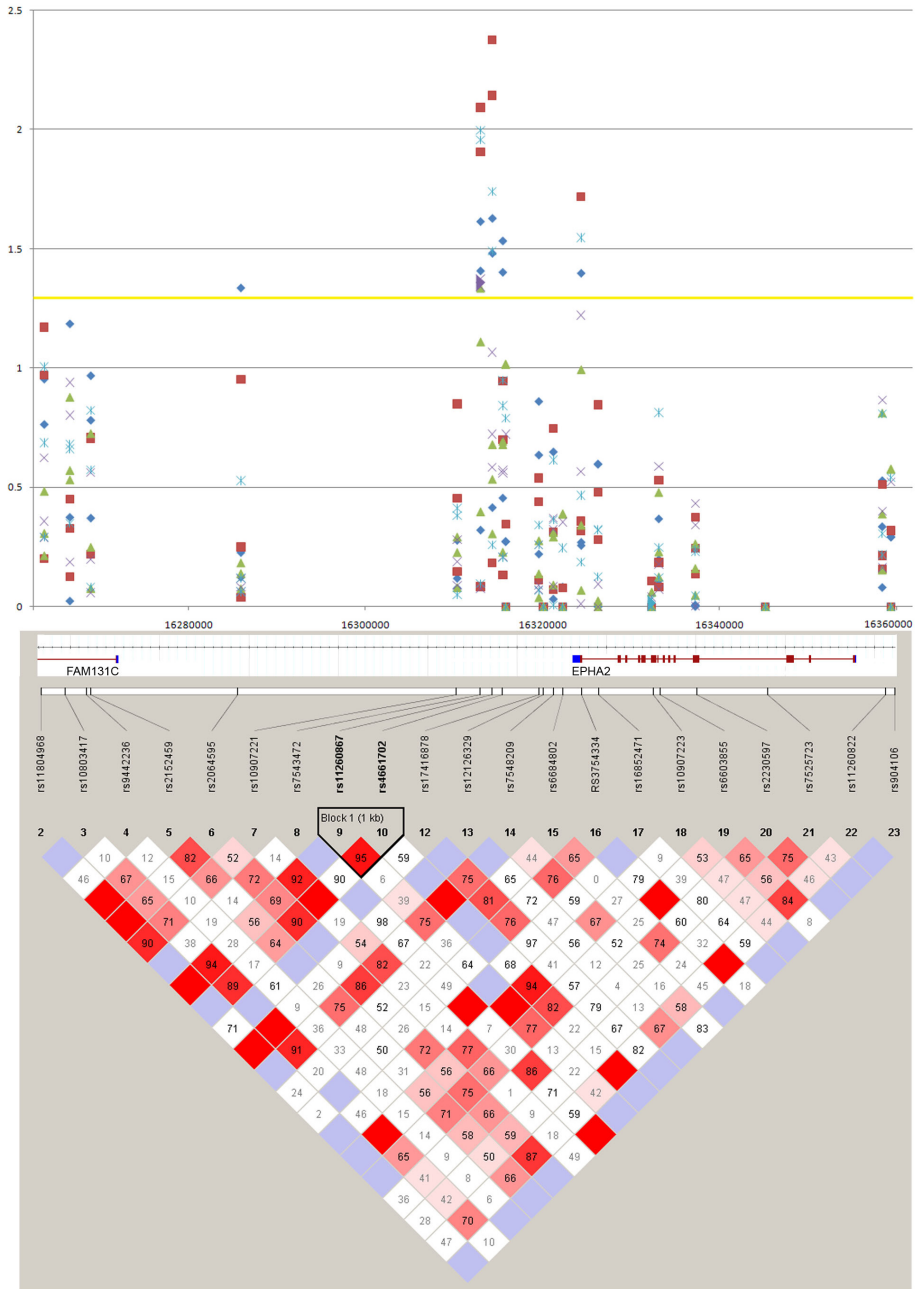
Graphical presentation of association between SNPs in the *EPHA2* region of chromosome 1p with age-related cataracts in the Italian case-control cohort. The upper panel shows a plot of -log p-values (y-axis) from association analyses of 21 SNPs across the *EPHA2* region. Blue diamonds denote -log p values for pure cortical cataracts; red squares, any cortical cataracts; green triangles, pure nuclear cataracts; purple x, any nuclear cataracts; and light blue asterisks, any cataracts. The x-axis shows the relative physical location of each SNP measured in mega-base-pairs. The lower panel shows a pairwise linkage disequilibrium (D´) Haploview plot for SNPs in the *EPHA2* region. The strength of linkage disequilibrium (LD) is color-coded; red indicates strong LD with SNPs showing high correlation, and blue indicates low LD and high recombination. The relative positions of *EPHA2* and the adjacent *FAM131C* are indicated with haplotype blocks for the European population (CEU) from the HapMap project (SNPbrowser, Applied Biosystems).

In an initial screen, tagging SNPs covering the *EPHA2* region were genotyped using 100 samples each from individuals with any nuclear, any cortical and no age-related cataracts. Markers yielding p values <0.05 were further analyzed using the entire complement of samples (Appendix 1, Figure 5). All markers were in Hardy–Weinberg equilibrium (HWE, p>0.05) for all sample groups tested. The highest levels of association occurred with rs7543472 (allelic p<0.038 for cortical cataracts, and allelic p<0.021 for any age related cataracts) and rs11260867 (allelic p<0.020 for cortical cataracts), which lie just distal (3′) to *EPHA2* (Figure 5). However, there was no significant association with nuclear cataracts, although results with rs7543472 were suggestive (allelic p<0.071, Appendix 1). These findings were true whether the analysis was for pure cortical cataracts, any cortical cataracts, pure nuclear cataracts, or any nuclear cataracts. Since association was seen between SNPs in the *EPHA2* region and cortical cataracts and suggestive results were obtained with nuclear cataracts, the analysis was extended to include any age-related cataracts with subsequent increased association in SNPs from the peak region to p<0.021 with the T-allele of rs7543472, although the G-allele of rs11260867 actually lost significance slightly (p=0.076). Results for SNPs outside this region changed only minimally. Because the SNPs showing the greatest association lay over or just beyond exon 17, this region was sequenced in all samples. The only variation occurring with a frequency greater than 0.05 was rs3754334, a synonymous p.I958I (C3011T) variant. This variant had minor allele frequencies of ~34% in cataract cases and 28% in controls, and was thus analyzed further, although the association was not as strong as with the two distal (3′) SNPs (rs7543472 or rs11260867) and no significant allelic p values were obtained. Allelic odds ratios ranged from 1.5 to 1.7, for rs7543472, and 1.2–1.9 for rs11260867, but were not significant for rs3754334. The p values obtained were not corrected for multiple testing as these SNPs were tested, a priori, for association with age-related cataracts on the basis of their close proximity to a gene linked with inherited cataracts that also maps within a known linkage region for age-related cataracts, rather than as part of a genome-wide scan.

Trend p values were very similar to allelic p values for all SNPs, but the specific genotypes show greater levels of association for all SNPs showing significant association (Appendix 1). The most significant was p<0.007 for cortical cataracts with rs11260867 (GG genotype), p<0.012 for cortical cataracts and p<0.01 for any cataracts with rs7543472 (TT genotype), and p<0.019 for cortical cataracts and p<0.028 for any cataracts with rs3754334 (TT genotype). In addition, association of nuclear cataracts with the TT genotype of rs7543472 is marginally significant at p<0.042 and suggestive with the TT genotype of rs3754334 at p<0.06.  Genotypic odds ratios show the greatest risk increase in individuals homozygous for the risk alleles, ranging from 1.9 to 2.1 for rs7543472, 1.5–2.3 for rs11260867, and 2.7–3.3 for rs3754334, depending on the type of age-related cataracts being considered (Appendix 1).

The association of a potential risk haplotype for these three SNPs was examined; however, the association level was found to be essentially that of the least associated SNP. This result was consistent with the observation that all but 4 affected individuals with the homozygous TT genotype for rs3754334 also had a homozygous TT genotype for rs7543472, and all but one had a homozygous GG genotype for rs11260867. Similarly, of affected individuals with a homozygous TT genotype for rs7543472 only a single individual did not have a homozygous GG genotype for rs11260867. However, because the genotype frequency of TT for rs3754334 was only ~15% it did not show strong linkage disequilibrium with rs11260867 (D´=0.018) or rs7543472 (D´=0.022). In contrast, rs11260867 and rs7543472, for which the risk genotypes have frequencies of ~80% and ~73% respectively, showed strong linkage disequilibrium (D´=0.896).

## Discussion

In this study, we have mapped an interval on human chromosome 1p36 for autosomal dominant posterior polar cataracts, and identified an underlying missense mutation (p.G948W) in the gene coding for EPHA2, which has not previously been associated with eye disease. Our disease interval maps in close proximity to autosomal dominant loci for the Volkman congenital cataract (CCV) in a Danish family [45], posterior polar cataract (CCTP1) in a British family [46], and total congenital cataract in a Tasmanian family [47], raising the possibility of allelism (Figure 1C). In addition, *EPHA2* resides within a large interval (D1S468-[~25.5 Mb]-D1S1622) on chromosome 1p (Figure 1C) that was previously linked with age-related cortical cataracts in families and sib-pairs from Beaver Dam, Wisconsin [44]. Here, we further demonstrate that variations in the *EPHA2* region are associated with age-related cortical cataracts (p=0.007), and age-related cataracts overall (p=0.01) in an Italian population.

*EPHA2* encodes a 976 amino acid, type-1 transmembrane protein (~108 kDa) with extracellular NH_2_-terminal and cytoplasmic COOH-terminal halves (Figure 4B) [42]. The extracellular region comprises a conserved Eph-ligand binding domain followed by a cysteine-rich EGF-like domain, and two fibronectin type-III repeats, whereas, the cytoplasmic region contains a conserved tyrosine kinase domain, and a sterile-α-motif (SAM) domain [42]. The proposed p.G948W missense substitution, cosegregating with cataracts in family Mu, was predicted to reside in the cytoplasmic SAM domain of EPHA2. The evolutionarily conserved ~70 amino acid SAM domain has a compact helical structure that is believed to facilitate protein–protein interactions [43], raising the possibility that the predicted p.G948W substitution may interfere with Eph-receptor oligomerization and clustering into higher-order complexes essential for physiologic signaling [42].

In contrast to the heterozygous genotype and dominant phenotype linked with the p.G948W mutation in *EPHA2*, the SNPs in the *EPHA2* region most associated with age-related cataracts displayed the highest odds ratios when homozygous for the risk allele, consistent with recessive effects. Furthermore, the p.G948W mutation was linked with posterior polar cataracts, which most resemble age-related posterior sub-capsular cataracts, whereas, *EPHA2* SNPs were associated with much more prevalent forms of age-related cataracts, in decreasing order of significance; cortical opacities > any opacities > nuclear opacities. While it is problematic to compare inherited and age-related forms of cataracts, it is noteworthy that some of the affected members of family Mu also developed nuclear and anterior polar opacities. These observations raise the possibility that *EPHA2* variants may contribute to a common pathogenic mechanism resulting in different cataract phenotypes. The existence of any such mechanism remains to be determined; however, it is interesting that the two SNPs in the *EPHA2* region most associated with age-related cataracts (rs7543472 and rs11260867) lie outside the coding region near the 3′-end of the gene, which has been shown to harbor highly conserved translational control sequences that are believed to facilitate an RNA-based post-transcriptional mechanism for localized regulation of gene expression within cells [48,49].

EPHA2 is a member of the largest sub-family of receptor tyrosine kinases, which are divided into 2 classes; the A-subclass comprising 10 receptor genes (*EPHA1–10*) and the B-sub-class with 6 receptor genes (*EPHB1–6*) [50,51]. Eph receptors interact with their cognate membrane-anchored ligands, referred to as ephrins (*EFNA1–6*, *EFNB1–3*), at cell-cell contact sites to activate bidirectional signaling pathways effecting diverse physiologic processes including; cell adhesion, repulsion, morphology, and migration [50,51]. Intriguingly, EPHA2 has been implicated in tumor angiogenesis and neurite outgrowth; however, little is known about its role in the development and homeostasis of the avascular, noninnervated lens. Recently, however, it has been reported that mice lacking ephrin-A5, a ligand of Epha2, develop cataracts as a result of impaired lens fiber cell adhesion [52]. Further characterization of Eph-ephrin signaling pathways in the lens should provide insight into the pathogenetic mechanisms linking EPHA2 dysfunction with cataractogenesis.

In conclusion, our data have associated *EPHA2* with both inherited and age-related cataracts, and suggest that dysfunction of a member of the EphA-receptor tyrosine kinase sub-family triggers loss of lens transparency.

## Appendix 1. Association results for SNPs in the EPHA2 region with age-related cataracts.

Calculated significance values (p=< 0.05) show most association between rs11260867 and any cortical cataracts, and between rs7543472 and any cataracts. To access the data, click or select the words “Appendix 1.” This will initiate the download of a compressed (pdf) archive that contains the file.

## References

[1] World Health Organization - Vision 2020; The Right to Sight, Global initiative for the elimination of avoidable blindness - Action plan 2006–2011 (http://www.who.int/blindness/Vision2020%20-report.pdf)

[2] SanGiovanni JP (2002). Infantile cataract in the collaborative perinatal project: prevalence and risk factors.. Arch Ophthalmol.

[3] Zetterstrom C (2005). Cataracts in children.. J Cataract Refract Surg.

[4] Shiels A (2007). Genetic origins of cataract.. Arch Ophthalmol.

[5] Litt M (1998). Autosomal dominant congenital cataract associated with a missense mutation in the human alpha crystallin gene CRYAA.. Hum Mol Genet.

[6] Berry V (2001). Alpha-B crystallin gene (CRYAB) mutation causes dominant congenital posterior polar cataract in humans.. Am J Hum Genet.

[7] Mackay DS (2002). A nonsense mutation in CRYBB1 associated with autosomal dominant cataract linked to human chromosome 22q.. Am J Hum Genet.

[8] Litt M (1997). Autosomal dominant cerulean cataract is associated with a chain termination mutation in the human beta-crystallin gene CRYBB2.. Hum Mol Genet.

[9] Riazuddin SA (2005). Mutations in betaB3-crystallin associated with autosomal recessive cataract in two Pakistani families.. Invest Ophthalmol Vis Sci.

[10] Kannabiran C (1998). Autosomal dominant zonular cataract with sutural opacities is associated with a splice mutation in the betaA3/A1-crystallin gene.. Mol Vis.

[11] Billingsley G (2006). CRYBA4, a novel human cataract gene, is also involved in microphthalmia.. Am J Hum Genet.

[12] Heon E (1999). The gamma-crystallins and human cataracts: a puzzle made clearer.. Am J Hum Genet.

[13] Sun H (2005). Gamma-S crystallin gene (CRYGS) mutation causes dominant progressive cortical cataract in humans.. J Med Genet.

[14] Shiels A (1998). A missense mutation in the human connexin50 gene (GJA8) underlies autosomal dominant “zonular pulverulent” cataract, on chromosome 1q.. Am J Hum Genet.

[15] Mackay D (1999). Connexin46 mutations in autosomal dominant congenital cataract.. Am J Hum Genet.

[16] Bu L (2002). Mutant DNA-binding domain of HSF4 is associated with autosomal dominant lamellar and Marner cataract.. Nat Genet.

[17] Jamieson RV (2002). Domain disruption and mutation of the bZIP transcription factor, MAF, associated with cataract, ocular anterior segment dysgenesis and coloboma.. Hum Mol Genet.

[18] Semina EV (1998). A novel homeobox gene PITX3 is mutated in families with autosomal-dominant cataracts and ASMD.. Nat Genet.

[19] Berry V (2000). Missense mutations in MIP underlie autosomal dominant 'polymorphic' and lamellar cataracts linked to 12q.. Nat Genet.

[20] Pras E (2002). A missense mutation in the LIM2 gene is associated with autosomal recessive presenile cataract in an inbred Iraqi Jewish family.. Am J Hum Genet.

[21] Jamieson RV (2007). Characterization of a familial t(16;22) balanced translocation associated with congenital cataract leads to identification of a novel gene, TMEM114, expressed in the lens and disrupted by the translocation.. Hum Mutat.

[22] Conley YP (2000). A juvenile-onset, progressive cataract locus on chromosome 3q21-q22 is associated with a missense mutation in the beaded filament structural protein-2.. Am J Hum Genet.

[23] Ramachandran RD (2007). Autosomal recessive juvenile onset cataract associated with mutation in BFSP1.. Hum Genet.

[24] Shiels A (2007). CHMP4B, a novel gene for autosomal dominant cataracts linked to chromosome 20q.. Am J Hum Genet.

[25] Kloeckener-Gruissem B (2008). Mutation of solute carrier SLC16A12 associates with a syndrome combining juvenile cataract with microcornea and renal glucosuria.. Am J Hum Genet.

[26] Heiba IM (1993). Genetic etiology of nuclear cataract: evidence for a major gene.. Am J Med Genet.

[27] Hammond CJ (2000). Genetic and environmental factors in age-related nuclear cataracts in monozygotic and dizygotic twins.. N Engl J Med.

[28] Congdon N (2004). Nuclear cataract shows significant familial aggregation in an older population after adjustment for possible shared environmental factors.. Invest Ophthalmol Vis Sci.

[29] Heiba IM (1995). Evidence for a major gene for cortical cataract.. Invest Ophthalmol Vis Sci.

[30] Hammond CJ (2001). The heritability of age-related cortical cataract: the twin eye study.. Invest Ophthalmol Vis Sci.

[31] Congdon N (2005). Cortical, but not posterior subcapsular, cataract shows significant familial aggregation in an older population after adjustment for possible shared environmental factors.. Ophthalmology.

[32] Hejtmancik JF (2004). Molecular genetics of age-related cataract.. Exp Eye Res.

[33] Okano Y (2001). A genetic factor for age-related cataract: identification and characterization of a novel galactokinase variant, “Osaka,” in Asians.. Am J Hum Genet.

[34] Maraini G (2003). Galactokinase gene mutations and age-related cataract. Lack of association in an Italian population.. Mol Vis.

[35] Clinical Trial of Nutritional Supplements and Age-Related Cataract Study Group (2008). A randomized, double-masked, placebo-controlled clinical trial of multivitamin supplementation for age-related lens opacities. Clinical trial of nutritional supplements and age-related cataract report no. 3.. Ophthalmology.

[36] Age Related Eye Disease Study Group (2001). The age-related eye disease study (AREDS) system for classifying cataracts from photographs: AREDS report no. 4.. Am J Ophthalmol.

[37] Hoffmann K (2005). easyLINKAGE Plus – Automated linkage analyses using large scale SNP data.. Bioinformatics.

[38] Mackay DS (2003). Cell death triggered by a novel mutation in the alphaA-crystallin gene underlies autosomal dominant cataract linked to chromosome 21q.. Eur J Hum Genet.

[39] Lathrop GM (1984). Strategies for multilocus linkage analysis in humans.. Proc Natl Acad Sci USA.

[40] Barrett JC (2005). Haploview: analysis and visualization of LD and haplotype maps.. Bioinformatics.

[41] Wistow G (2008). NEIBank: genomics and bioinformatics resources for vision research.. Mol Vis.

[42] Himanen JP (2003). Eph signaling: a structural view.. Trends Neurosci.

[43] Stapleton D (1999). The crystal structure of an Eph receptor SAM domain reveals a mechanism for modular dimerization.. Nat Struct Biol.

[44] Iyengar SK (2004). Identification of a major locus for age-related cortical cataract on chromosome 6p12-q12 in the Beaver Dam Eye Study.. Proc Natl Acad Sci USA.

[45] Eiberg H (1995). Assignment of congenital cataract Volkmann type (CCV) to chromosome 1p36.. Hum Genet.

[46] Ionides AC (1997). A locus for autosomal dominant posterior polar cataract on chromosome 1p.. Hum Mol Genet.

[47] McKay JD (2005). The telomere of human chromosome 1p contains at least two independent autosomal dominant congenital cataract genes.. Br J Ophthalmol.

[48] Brittis PA (2002). Axonal protein synthesis provides a mechanism for localized regulation at an intermediate target.. Cell.

[49] Winter J (2008). Comparative 3′UTR analysis allows identification of regulatory clusters that drive Eph/ephrin expression in cancer cell lines.. PLoS One.

[50] Pasquale EB (2005). Eph receptor signaling casts a wide net on cell behaviour.. Nat Rev Mol Cell Biol.

[51] Pasquale EB (2008). Eph-ephrin bidirectional signaling in physiology and disease.. Cell.

[52] Cooper MA (2008). Loss of ephrin-A5 function disrupts lens fiber cell packing and leads to cataract.. Proc Natl Acad Sci USA.

